# High-Intensity Interval Training Improves Cardiac Autonomic Function in Patients with Type 2 Diabetes: A Randomized Controlled Trial

**DOI:** 10.3390/biology11010066

**Published:** 2022-01-02

**Authors:** Lucas Raphael Bento Silva, Paulo Gentil, Camila Simões Seguro, Jordana Campos Martins de Oliveira, Maria Sebastiana Silva, Vitor Alves Marques, Thomas Beltrame, Ana Cristina Silva Rebelo

**Affiliations:** 1 Department of Physical Education, Araguaia University Center, Goiania 74223-060, Brazil; jordana.oliveira@uniaraguaia.edu.br; 2 Post-Graduate Program in Health Sciences, Faculty of Medicine, Federal University of Goias, Goiania 74605-050, Brazil; paulogentil@ufg.br (P.G.); vitor_alvesmarques@hotmail.com (V.A.M.); ana_rebelo@ufg.br (A.C.S.R.); 3 Faculty of Physical Education and Dance, Federal University of Goiás, Goiania 74690-900, Brazil; mssilva@ufg.br; 4 Hypertension League, Federal University of Goias, Goiania 74605-020, Brazil; 5 Post-Graduate Program in Nutrition and Health, Faculty of Nutrition, Federal University of Goias, Goiania 74605-080, Brazil; camila.simoes@discente.ufg.br; 6 Institute of Computing, University of Campinas, Campinas 13083-852, Brazil; beltramethomas@gmail.com; 7 Department of Physiotherapy, Federal University of Sao Carlos, Sao Carlos 13565-905, Brazil; 8 Department of Morphology, Institute of Biological Sciences, Federal University of Goiás, Goiania 74690-900, Brazil

**Keywords:** type 2 diabetes, physical exercise, high-intensity interval training, cardiac autonomic modulation, heart rate recovery, heart rate variability, aerobic training, health

## Abstract

**Simple Summary:**

Diabetes mellitus is a metabolic disorder characterized by an increased blood glucose concentration. The most common diabetes is type 2, corresponding to approximately 95% of the diagnosed cases. Chronic hyperglycemia can lead to many complications such as increased incidence of cardiovascular diseases as well as renal and ophthalmologic complications. Physical exercise is seen as an effective non-pharmacological strategy for managing the disease. In the present study, 44 middle-aged adults with type 2 diabetes were recruited and stratified into three exercise groups: HIIT-30:30, HIIT-2:2, and MICT. All patients were submitted to anamnesis, evaluation of cardiorespiratory fitness, and cardiac autonomic modulation, and were submitted to physical exercise programs for eight weeks. From the results found, it was possible to infer that high intensity physical training programs can be safe and effective for patients with type 2 diabetes and might be performed in different phases of a rehabilitation program. However, it is necessary to know how to work with the prescription of these exercises considering its cost effectiveness, because, in this study, the protocols HIIT-2:2 and HIIT-30:30 presented superior benefits to the MICT protocol.

**Abstract:**

Different exercise models have been used in patients with type 2 diabetes mellitus (T2D), like moderate intensity continuous training (MICT) and high intensity interval training (HIIT); however, their effects on autonomic modulation are unknown. The present study aimed to compare the effects of different exercise modes on autonomic modulation in patients with T2D. In total, 44 adults with >5 years of T2D diagnosis were recruited and stratified into three groups: HIIT-30:30 (*n* = 15, age 59.13 ± 5.57 years) that performed 20 repetitions of 30 s at 100% of VO2peak with passive recovery, HIIT-2:2 (*n* = 14, age 61.20 ± 2.88) that performed 5 repetitions of 2 min at 100% of VO2peak with passive recovery, and MICT (*n* = 15, age 58.50 ± 5.26) that performed 14 min of continuous exercise at 70% of VO2peak. All participants underwent anamnesis and evaluation of cardiorespiratory fitness and cardiac autonomic modulation. All protocols were equated by total distance and were performed two times per week for 8 weeks. Group × time interactions were observed for resting heart rate (HRrest) [F(2.82) = 3.641; *p* = 0.031] and *SDNN* [F(2.82) = 3.462; *p* = 0.036]. Only the HIIT-30:30 group significantly reduced *SDNN* (*p* = 0.002 and 0.025, respectively). HRrest reduced more in the HIIT-30:30 group compared with the MICT group (*p* = 0.038). Group × time interactions were also observed for offTAU [F(2.82) = 3.146; *p* = 0.048] and offTMR [F(2.82) = 4.424; *p* = 0.015]. The MICT group presented increased values of offTAU compared with the HIIT-30:30 and HIIT-2:2 groups (*p* = 0.001 and 0.013, respectively), representing a slower HR response after eight weeks of intervention. HIIT, specially HIIT-30:30, represents a promising measure for improving autonomic modulation in patients with T2D.

## 1. Introduction

Projections estimate that, by 2045, the number of people diagnosed with diabetes will reach 693 million worldwide [1]. Type 2 diabetes (T2D) represents 90–95% of diagnoses, is more prevalent in adults over 40 years old, and can be caused by environmental (association with risk factors, such as obesity and sedentary lifestyle) or genetic factors [2]. T2D is associated with an increased risk of mortality, especially oeing to cardiovascular diseases [3].

Among the many problems in the cardiovascular system associated with T2D [4], there seems to be an association between chronic hyperglycemia and changes in cardiovascular autonomic nervous system, as verified by heart rate variability (HRV) indexes [5,6]. In previous studies, our research group showed attenuation of the response of the heart rate recovery (HRR) in patients with T2D and an association between blood glucose levels and the slowed response observed from the amplitude (Amp) indexes, which reflects the angulation of the heart rate (HR) curve after interruption of physical exercise and the tau time constant (τ), representing the time of HR decay in adults with T2D [7].

The association between cardiac autonomic modulation and cardiovascular events, as well as all-cause mortality, in the general population has been described in the current literature; individuals with low values for HRV and HRR present a higher risk of some severe cardiovascular outcome, which seems to associated with a deficiency in response to physiological stress [3,8]. Thus, it seems important to evaluate these variables in populations with different clinical conditions and under stress.

The importance of physical exercise as a non-pharmacological measure for the prevention of T2D [9] has been increasingly acknowledged, and physical exercise may decrease the risk of T2D and its cardiovascular complications [10,11]. Moderate aerobic physical training is related to numerous beneficial effects on glycemic control [12,13] and positive clinical outcomes in individuals with T2D, including a reduction in glycated hemoglobin (Hb1Ac) [14,15], increased oxygen consumption (VO2peak) [16], and an improvement in insulin sensitivity [17]. However, in recent decades, studies revealed that high-intensity interval training (HIIT) has many benefits and might be a good time-efficient approach for exercise [18,19,20]. However, HIIT might be performed in many different ways, with different combinations of effort and recovery, which might affect its acute and chronic responses [21,22,23,24].

Previous studies evaluated the acute effects of three exercise modes in healthy young people [25,26], the protocols tested were based on the intensity at which the VO2max was achieved (WLVO2max): HIIT-4:3 (3 repetitions of 4 min at 90% of WLVO2max and 3 min recovery at 60% of WLVO2max), HIIT-30:30 (29 repetitions of 30 s at 100% of WLVO2max and 30 s of passive recovery between), and MICT (continuous intensity for 21 min at 70% of WLVO2max). According the results, HIIT-4:3 resulted in a higher heart rate and increased ratings of perceived exertion. Moreover, HIIT-4:3 and MICT promoted slower HRR responses when compared with HIIT-30:30, and both also showed exacerbation of sympathetic modulation after physical exercise in HRV measurements. These results suggested that performing longer bouts during HIIT might impose greater cardiovascular stress, while HIIT with sorter bouts might be safer. However, interventions with long-term exercise, as well as their responses in individuals with T2D, require further elucidation. 

It is important to compare different types of physical exercise to identify interventions that promote better physiological adaptations and have a better understanding of their cost–benefit. Therefore, the aim of this study was to evaluate the effects of different modes of physical exercise on cardiac autonomic modulation in T2D patients.

## 2. Materials and Methods

### 2.1. Study Design

The present study is characterized as an experimental, randomized clinical trial. Sixty patients were selected for the initial tests. The following inclusion criteria were employed: (1) age between 50 and 65 years, (2) diagnosed with T2D for at least five years, and (3) not being involved with regular physical activity over the last 6 months. 

Tests were performed in four stages: cardiopulmonary exercise test (CPET) and cardiac autonomic modulation. After CPET, eight patients were excluded, including three with an exercise capacity <6 METS, two with uncontrolled arrhythmias during physical exertion, one with unstable angina, and two with a reduction in SBP less than that of SBP levels at rest and during exercise. After the exclusion of ineligible participants, participants were randomized into three groups using the website www.random.org (accessed on 1 November 2021).

The characteristics of the study participants who were stratified into three groups, including HIIT-30:30 (*n* = 15), HIIT-2:2 (*n* = 14), and MICT (*n* = 15), are shown in Table 1. Six participants did not reach minimal attendance of >85% and were excluded from the analysis.

**Figure 1:**
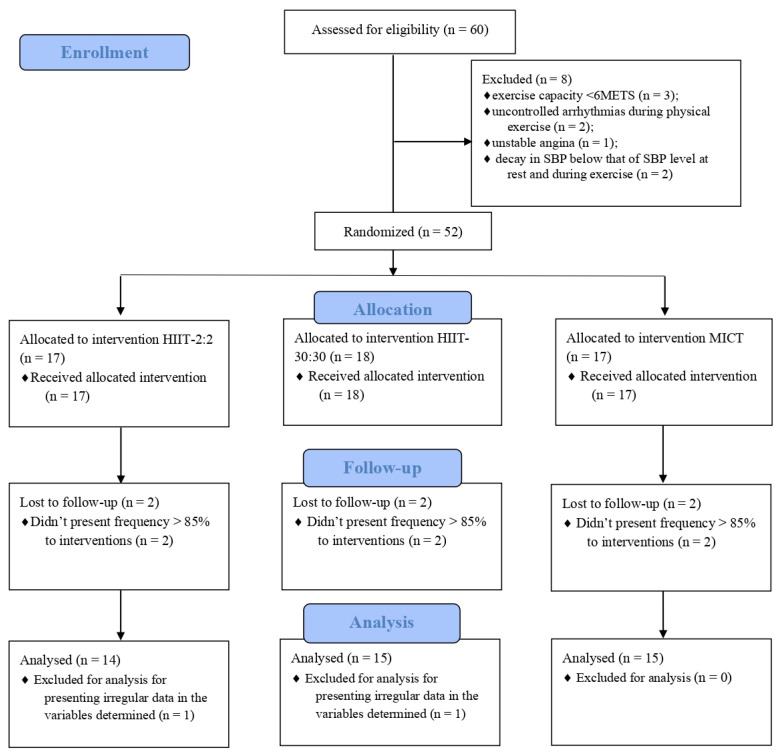
Study flow according to CONSORT recommendations. MICT: moderate intensity continuous training; HIIT: high-intensity interval training; SBP: sistolic blood pressure.

Two patients were excluded because they presented irregular data for the analyses of the determined variables, as shown in Figure 1. The project was approved by the research ethics committee (CEP) of the Institution under Opinion No. 2,667,732 and CAAE No. 54522016.6.0000.5083 and duly registered in the Brazilian Registry of Clinical Trials (ReBEC) under number TRIAL: RBR-4RJGC3.

### 2.2. Test Protocols

**Figure 2:**
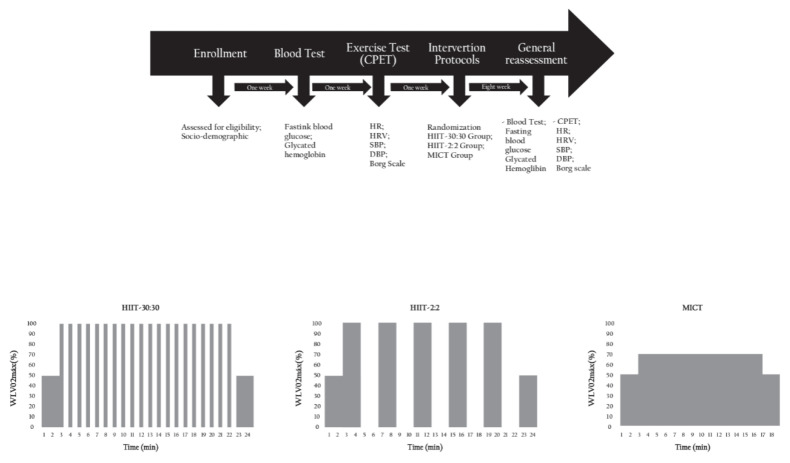
Study design. HR: heart rate; HRV: heart rate variability; SBP: systolic blood pressure; DBP: diastolic blood pressure.

The participants made four visits to the laboratories. The first visit occurred to explain the study and familiarise the participants with the procedures and equipment used. In the second visit, blood tests were conducted to confirm the diagnosis of T2D. In the third visit, cardiorespiratory fitness and cardiac autonomic modulation were evaluated. General reassessments were performed after eight weeks of intervention, as shown in Figure 2.

Postmenopausal women who reported drug use for hormone replacement therapy for at least 12 months were included in the study and evaluated during the placebo phase of the medication [6].

#### 2.2.1. Cardiopulmonary Exercise Test (CPET)

Volunteers were instructed to wear comfortable clothes and avoid vigorous physical exercise (for 24 h before the test), alcohol intake (12 h before the test), and caffeine intake (3 h before the test). All CPETs were performed in the morning to avoid the influence of circadian rhythm on the studied variables. Room temperature (22–24 °C), relative humidity (40–60%), and lighting were controlled according to the preliminary conditions to ensure consistent evaluations. The volunteers were informed regarding protocols, rating of perceived exertion (RPE) scale, and the criteria for interrupting the test.

The ramp-type load increment protocol was applied with a total duration of eight to 12 min. Each volunteer started the test with a warm-up and adaptation for two minutes at 50% speed of the initial values predicted for age and sex. The speed was increased by 0.5 km/h for every 15 s of warm-up.

During the test, the initial treadmill speeds were previously programmed according to age and gender, following the recommendations of the Brazilian Society of Cardiology (SBC). The velocity increased 0.1 km/h every 10, 20, or 30 s, and there was no increase in inclination, as the protocols were executed without inclination.

The active recovery period lasted four minutes at 0% inclination and 50% of the maximum speed reached, and the speed decreased by 10% every 30 s.

Cardiorespiratory fitness was directly assessed through the CPET. The treadmill (Micromed®, Centurion 200, Brasília, Brazil) was coupled to a computer for data processing. Gas analysis was performed using the Cortex analyser® (Metalyser II, Rome, Italy). The calibration of the equipment was performed for barometric pressure, ambient gas, gas mixture, flow, and volume according to the manufacturer’s recommendations. During the CPET protocol, data on HR, blood pressure (SBP and DBP), subjective perception of exertion, and ventilatory parameters were collected.

HR was continuously monitored using a heart monitor (Polar v800, Oulu, Finland). Blood pressure was measured by Korotkoff auscultation with a mercury sphygmomanometer (WanMed, São Paulo, SP, Brazil) and a stethoscope (Littman, São Paulo, Minnesota, USA) in the following positions: supine, sitting, and pre-CPET. To assess the subjective perception of exertion, the Borg scale was used [27].

The criteria for the interruption of CPET were recommended by the American College of Sports Medicine [28] as follows:

(1) discontinuity of stride during the treadmill running phase;
(2) reaching the predicted maximum heart rate (HRmax) for the patient’s age;
(3) respiratory exchange ratio > 1.15.

#### 2.2.2. Heart Rate Variability (HRV)

Volunteers were instructed to sit and rest for 10 min to obtain a reliable baseline R-Ri recording. Then, a heart rate monitor (Polar v800, Finland) attached to the chest was used to record the 10-min R–R interval between the rest and supine positions, which was transmitted to the heart monitor in real time.

Subsequently, the data were transferred to Microsoft Excel^®^ (2016 version, Redmond, WA, USA) to remove artifacts. After recording the HRV at rest, the data were transferred to the portable microcomputer via a USB cable. The data obtained were processed by the Kubios HRV version 3.2 software (Kuopio, Finland). Average correction filters and visual data analysis were used for error detection and correction.

For time domain analysis, the *RMSSD* (square root of the square mean of the differences between adjacent normal *RR* intervals in a time interval, expressed in ms) was used as a marker of parasympathetic, and *SDNN* indices (standard deviation of all normal *RR* intervals recorded in a time interval, expressed in ms) were used as the global HRV index. Further, the variable PNN50 represents the percentage of R–R intervals with variation greater than 50 ms, which also corresponds to the vagal modulation of the individual. *SDDN* and rMSSD were calculated according to the following formulas:

(1) $$SDNN=\sqrt{\frac{1}{N-1}\overset{N}{\underset{j=1}{\sum }}{\left(RR_{j}-\bar{RR}\right)}^{2}}$$

(2) $$RMSSD=\sqrt{\frac{1}{N-1}\overset{N-1}{\underset{j=1}{\sum }}{\left(RR_{j+1}-RR_{j}\right)}^{2}}$$

where $RR_{j}$ corresponds to the value of the *j*-th *RR* interval, *N* represents the total number of successive intervals, and $\bar{RR}$ represents the average of the *RR* intervals.

#### 2.2.3. Symbolic Analysis and Shannon’s Entropy 

Symbolic analysis was performed by dividing all possible symbolic patterns into four categories: (I) patterns are the same and there is no change (0V, such as 2–2–2 or 4–4–4 or 5–5–5); (II) a single change in the pattern (1V, which is 2–2–3 or 4–2–2); (III) two similar forms of change in the pattern that form ascending or descending lines (2LV, which is 5–3–1 or 1–2–4); and (IV) two distinct variations, in which three symbols form a peak or valley (2UV, such as 1–4–2 or 5–2–4), being evaluated according to the frequency of occurrence (0V%, 1V%, 2LV%, and 2UV%). Previous studies have evaluated that the 0V family represents only sympathetic modulation, 1V represents parasympathetic and sympathetic modulations, and 2LV also represents both parasympathetic and sympathetic modulations being dominated by the valgus nerve. Finally, the 2UV family only represents parasympathetic modulation [29].

Shannon entropy (SE) reflects the complexity of distributing these symbolic patterns. If the distribution is flat (the pattern is evenly distributed), the SE will be high and, when a subset of the patterns is unlikely, nonexistent, or rare, it will be very low [30].

#### 2.2.4. Monoexponential Analyses

The HRR data were obtained after the interruption of the CPET, and filtered and analyzed through a specific procedure developed in the OriginPro 8.0 software (OriginLab, Northampton, MA, USA) that applies an exponential model to the data for the entire recovery period (four minutes of active recovery and three minutes of passive recovery in the orthostatic position) [26,31].

To obtain the best parameters of this exponential curve, a nonlinear algorithm was used that adopts the minimization of the sum of square errors as a convergence criterion. Only the r > 0.95 function was included in the final analyses. The off kinetics was modulated using an exponential function of time as follows:

HR (t) = HRend − a*(1 − e ^−(t−TD)/t^) (3)

where “t” is time; "HRend" is the heart rate at the end of the CPET; “Amp” is the amplitude of HR decrease after the end of the exercise; and “TD” is the delay time for the function. The inclusion of the term “TD” in this function was established owing to the possibility that the HRR is not immediately reduced after the load interruption. Because the parameter “τ” is a time constant in a negative decreasing exponential function, it can be inferred that, the lower its value, the faster the kinetics of the HRR [32].

### 2.3. Exercise Protocols

The protocols were customized with individualized monitoring of HR and their respective exercise intensities were adapted from previous studies [33,34].

The participants performed 2 min of warm-up at 50% of WLVO2max. In the cool down, the participants performed 2 min of recovery at 50% of WLVO2max. In the HIIT-30:30 protocol, participants performed 20 bouts of 30 s at 100% of WLVO2max with 30 s of passive intervals. In the HIIT-2:2 protocol, the participants performed 5 bouts of 2 min at 100% of WLVO2max with passive rest intervals. In the MICT protocol, the participants exercise continuously for 14 min at 70% of WLVO2max, as shown in Figure 2. In each training session, SBP and DBP, HR, and glycemia values were measured and recorded before and 10 min after the end of the session.

### 2.4. Statistical Analyses

We performed an a priori calculation to estimate sample size using GPower 3.1.9.2 (Düsseldorf University, Düsseldorf, Germany). The parameters were as follows: 0.5 effect size (medium), 0.05 alfa level, 0.8 power, 3 groups, 6 measures, and 0.5 correlation among repeated measures. The results suggested a total sample size of 27 for between-group comparisons. However, considering the possible attrition and large number of volunteers, we accepted considerably more participants.

The Shapiro–Wilk test was used to evaluate the normality of the data, and Levene’s test was used to assess sample homogeneity. One-way ANOVA was used to verify the differences between the groups at baseline. Two-way ANOVA with repeated measurements was used to verify the inter-group differences. When these differences were found, the post hoc Ryan–Einot–Gabriel–Welsh Q (REGWQ) test was used. 

The effect-size for the samples was based on the calculation of the rank-biserial correlation (rB). The rB values were classified according to the classification criteria for Pearson’s correlation coefficient and, therefore, trivial (rB < 0.10), small (0.10 ≤ rB < 0.30), medium (0.30 ≤ rB < 0.50), and large (rB ≥ 0.50) [35].

A statistical analysis was performed using the statistical program Statistical Package for the Social Sciences (SPSS; Armonk, NY, USA; IBM Corp.), version 21. *p* < 0.05 was considered significant in all analyses.

## 3. Results

Table 2 presents the results of HR and HRV responses for each group. The two-way ANOVA revealed the effect of the intervention in the variables HRpeak [F(2.82) = 5.091; *p* = 0.009] and pNN50 [F(2.82) = 5.071; *p* = 0.008], with a significant increase observed only in the HIIT-30:30 group (medium effect for variables). Time effect was observed in the variables HRrest [F(1.82) = 7,097; *p* = 0.009], with a reduction observed for HIIT-30:30 group (r_B_ = 0.50—medium effect), R-Ri [F(1.82) = 0.045; *p* = 0.001], pNN50 [F (1.81) = 10.413; *p* = 0.002], and 2UV% [F(1.82) = 9.285; *p* = 0.003], with the HIIT-30:30 group presenting a postintervention increase (r_B_ = −0.24,small effect; −0.39,medium effect; and −0.50, medium effect, respectively).

A group × time interaction was observed for the variables HRrest [F(2.82) = 3.641; *p* = 0.031]; the HIIT-30:30 group exhibited a greater reduction in HRrest than the MICT group (*p* = 0.038), R-Ri [F(2.82) = 4.420; *p* = 0.015]; the post-hoc analysis showed that there was significant diffusion when comparing the MICT group to groups HIIT-30:30 and HIIT-2:2 (*p* = 0.007 and 0.047 , respectively); *SDNN* [F(2.82) = 3.462; *p* = 0.036] showed a significant reduction only in the HIIT-30:30 (*p* = 0.025) and 2UV% [F(2.82) = 3.708; *p* = 0.029] groups; and two groups submitted to high intensity protocols, HIIT-30:30 and HIIT-2:2, showed a significant increase in the variable 2UV% (*p* = 0.001 and 0.025, respectively), which reflects vagal modulation, as observed in the post-hoc analysis.

Table 3 shows the results obtained from the analyses of HR kinetics (off parameters) in the groups in the pre- and post-assessments. A group effect was found for offTAU [F(2.82) = 4.710; *p* = 0.012] and offTMR [F(2.82) = 6.667; *p* = 0.002]. A time effect was found for offAMP [F(1.82) = 4.881; *p* = 0.030]. A group × time interaction was also observed for offTAU [F(2.82) = 3.146; *p* = 0.048] and offTMR [F(2.82) = 4.424; *p* = 0.015]. The MICT group presented higher values of offTAU compared with the HIIT-30:30 and HIIT-2:2 groups (*p* = 0.001—medium effect and 0.013—medium effect, respectively) in the post-intervention, representing increased slowness in the HR response after physical exertion. The MICT group showed a significant increase (*p* = 0.012) in offTMR as a function of time, with greater values than both the HIIT-30:30 and HIIT-2:2 groups (*p* < 0.001—medium effect and 0.008—trivial effect, respectively), indicating a slower HR response time after interruption of physical exercise.

## 4. Discussion

The present study aimed to compare the effects of three different physical exercise programmes on cardiac autonomic modulation in patients with T2D. The main results of this study show that 8 weeks of HIIT (especially HIIT-30:30) presented better results in cardiac autonomic modulation compared with MICT. No significant differences were observed between the two HIIT programmes. It is difficult to compare our results with previous studies, as the vast majority of studies to date have chosen only the application of MICT in patients with T2D [36,37,38].

Previous studies have shown that increased HRrest is associated with all-cause mortality [39,40,41]. In particular, the study by Prasada et al [42] evaluated individuals with T2D and showed that an increase of one unit in the standard deviation of the HRrest is associated with a 20% increase in the risk of mortality from cardiovascular disease in patients with T2D. In this regard, HIIT-30:30 might be particularly interesting, as it promoted a significant reduction in this parameter, with a greater reduction than those observed in the MICT group. This corroborates previous studies that observed an improvement in HRrest in individuals undergoing HIIT protocols [43,44].

Regarding HRV, the HIIT-30:30 protocol exhibited a 69.7% improvement in the *SDNN* index, and this result was statistically superior to the other protocols. This is consistent with the previous study that followed individuals with metabolic syndrome and found a significant increase in *SDDN* index, especially in the group that realized the HIIT [45]. The HIIT-30:30 and HIIIT-2:2 groups showed increases of 63.05% and 46.42% in the 2UV% index, respectively, which were higher than those observed after MICT. Both HIIT groups showed a tendency to reduce the sympathetic modulation perceived through the 0V index. These suggest a positive autonomic cardiac adaptation after eight weeks of intervention with HIIT. 

In the present study, HIIT-30:30 and HIIT-2:2 resulted in a faster decrease in HRR after the CPET when compared with the MICT protocol. No significant differences were noted between the two HIIT programmes. The benefit of HIIT on HRR has been suggested by Dall et al [46] when comparing the responses of 12 weeks of different exercise intensities in cardiac transplant recipients. The HIIT group performed 16 min interval training, with intervals of 4, 2, and 1 min duration and intensity above 80% of VO2peak with active recovery and duration of 2 min with intensity close to 60% of VO2peak. The MICT group performed 45 min of training on a bicycle with intensity between 60% to 70% of VO2peak. The authors observed an improvement in HRR in both groups, with a trend of greater improvement for the group that performed HIIT.

In agreement with our findings, Villelabeitia-Jaureguizar et al. [47] submitted 73 patients with coronary heart disease to eight weeks of physical training. The participants were divided into two groups. The HIIT performed 20 s at 50% of the maximum load and recovery of 40 s to 10% of the maximum load obtained in a steep ramp test, with progressions in the number of repetitions each week. The MICT group performed continuous exercise with the load of the first threshold with progression in time throughout the program. According to the results, the HIIT group presented better values in post-intervention HRR when compared with MICT.

There is already consensus in the literature that an attenuated HRR response is associated with the risk of cardiovascular events and all-cause mortality in the general population; people who have slowing HRR are 68% more likely to be affected by some cardiovascular event and have a 69% greater chance of mortality when compared with those who have a rapid response in HRR [48,49].

In our study, participants who performed eight weeks of HIIT protocols obtained better responses in autonomic modulation and HR parameters when compared with those who performed the MICT protocol. The reasons for these differences are not completely known. However, it might be possible that high intensity exercises influence autonomic nervous system adjustments to the heart and blood vessels to mediate the increased cardiovascular responses and metabolic demands [50]. In this regard, previous studies suggested that dynamic interactions between feed-forward and feedback circuits of central command and exercise pressor reflex are important in determining the long-term adjustments in the sympathetic and parasympathetic nervous systems [50,51]. Considering that these demands are intensity-dependent [52], HIIT might have promoted superior acute stress, resulting in higher adaptation than MICT.

Based on our results, we believe that HIIT should be included as a part of the patients’ T2D routine in order to improve cardiac autonomic modulation. Future studies are suggested to evaluate the effects of other modes of HIIT, manipulating exercise types and volume and intensity of training, in order to define the optimal protocol.

### Limitations

A limitation of our study was the fact that we did not evaluate HRV for longer periods, such as 12 or 24 h after the physical exercise sessions, in order to verify the cardiovascular stress generated by the intensity of the applied protocol. Another limitation is the absence of nutritional control in the patients.

## 5. Conclusions

HIIT, especially the HIIT-30:30 protocol, promoted an increase in R-Ri, *SDNN*, pNN50, and 2UV%, in addition to reducing HRrest, compared with continuous moderate intensity training. With these findings, we can suggest that HIIT is a feasible tool and that it can be implemented in cardiovascular rehabilitation programs.

## Figures and Tables

**Table 1:** Characteristics, risk factors, and medications of the study volunteers.

Variables	HIIT-30:30 (*n* = 15)	HIIT-2:2 (*n* = 14)	MICT (*n* = 15)
Age, years	59.13 ± 5.57	61.20 ± 2.88	58.50 ± 5.26
Sex (M/F)	6/9	6/8	7/8
Fasting blood glucose	142.70 ± 63.01	145.47 ± 66.00	129.79 ± 57.12
HbA1c, %	9.62 ± 1.90	9.85 ± 2.79	8.50 ± 2.43
Diagnosis time	>5 years	>5 years	>5 years
Biguandas, %	80 (12)	73.33 (11)	71.42 (10)
Medicines for sulphonylurea, %	-	6.66 (1)	21.42 (3)
SGLT2 inhibitors, %	-	13.33 (2)	7.14 (1)
DPP-4 inhibitors, %	6.66 (1)	6.66 (1)	7.14 (1)
GLP-1 analogue, %	6.66 (1)	-	14.28 (2)
Pioglitazone, %	6.66 (1)	-	7.14 (1)
Insulin, %	46.66 (7)	53.33(8)	35.71 (5)
Hypertension, %	100 (15)	100 (15)	100 (14)
Beta-blockers, %	-	-	-
ACE inhibitors, %	46,66 (7)	35,71 (5)	28,57 (4)
Diuretics, %	53,33 (8)	64,28 (9)	46,66 (7)
Dyslipidemia, %	80 (12)	86.66 (13)	78.57 (11)

Percentage values are expressed as % (absolute number), M: male, F: female, HbA1c: glycated hemoglobin, SGLT-2: sodium glucose linked transporter 2, DPP-4: dipeptidil peptidase 4, GLP-1: glucagon-like peptide 1, ACE: angiotensin-stenin-sizing enzyme.

**Table 2:** Linear and symbolic analysis of heart rate variability in groups submitted to the intervention program.

Variables	HIIT-30:30 (*n* = 15)		ES	HIIT-2:2 (*n* = 14)		ES	MICT (*n* = 15)		ES	Group	Time	Group*Time
	Pre	Post		Pre	Post		Pre	Post				
HRrest, bpm	82.86 ± 9.64	72.13 ± 8.62	0.50_(medium)_	84.85 ± 10.28	77.92 ± 8.96	0.33_(medium)_	78.86 ± 8.56	80.73 ± 9.43	−0.10_(small)_	0.278	0.009	0.031
HRpeak, bpm	152.46 ± 13.42	160.20 ± 14.06	−0.56_(medium)_	152.00 ± 21.37	158.35 ± 19.26	−0.31_(medium)_	146.40 ± 14.27	141.26 ± 17.46	0.15_(small)_	0.009	0.408	0.274
R-Ri, ms	821.2 ± 147.55	905.0 ± 178.9	−0.24_(small)_	822.7 ± 73.55	866.2 ± 85.7	−0.26_(small)_	867.33 ± 105.0	718.0 ± 275.5	0.33_(medium)_	0.224	0.832	0.015
*Linear Anaysis*												
*SDNN*, ms	20.13 ± 9.67	33.80 ± 22.04	−0.37_(medium)_	23.45 ± 15.12	28.65 ± 18.08	−0.15_(small)_	29.22 ± 20.92	21.85 ± 9.11	0.22_(small)_	0.932	0.133	0.036
rMSSD, ms	23.63 ± 13.87	38.39 ± 27.76	−0.31_(medium)_	18.51 ± 9.73	25.07 ± 10.33	−0.31_(medium)_	29.22 ± 23.19	22.16 ± 13.89	0.18_(small)_	0.150	0.217	0.065
pNN50, %	1.92 ± 3.21	9.70 ± 12.31	−0.39_(medium)_	1.02 ± 1.97	3.76 ± 3.61	−0.42_(medium)_	6.00 ± 8.59	12.85 ± 12.75	−0.30_(medium)_	0.008	0.002	0.485
*Nonlinear Analysis*												
0V%	39.23 ± 25.05	28.20 ± 11.14	0.27_(small)_	41.89 ± 25.50	31.09 ± 14.65	0.25_(small)_	38.33 ± 20.98	47.59 ± 27.75	−0.18_(small)_	0.226	0.355	0.111
1V%	33.67 ± 14.32	33.78 ± 13.97	−0.00_(trivial)_	31.93 ± 16.75	36.41 ± 12.11	−0.15_(small)_	35.20 ± 12.99	27.41 ± 12.18	0.29_(small)_	0.692	0.718	0.234
2LV%	9.62 ± 9.82	9.54 ± 5.58	0.00_(trivial)_	8.12 ± 5.16	8.37 ± 6.25	−0.02_(trivial)_	7.71 ± 7.37	7.05 ± 7.15	0.04_(trivial)_	0.482	0.918	0.970
2UV%	17.46 ± 10.72	28.47 ± 7.88	−0.50_(medium)_	16.78 ± 9.64	24.57 ± 7.00	−0.41_(medium)_	18.96 ± 8.21	17.73 ± 9.96	0.06_(trivial)_	0.146	0.003	0.029
SE	2.90 ± 1.29	3.18 ± 1.14	−0.11_(small)_	2.65 ± 1.38	2.94 ± 0.90	−0.12_(small)_	3.14 ± 1.01	2.68 ± 1.02	0.22_(small)_	0.724	0.888	0.380

HIIT: high intensity interval training, MICT: moderate continuous intensity training, Group*Time: corresponds to the group x time interaction, HRrest: resting heart rate in supine position, HRpeak: heart rate at peak physical exercise, iR–R: intervals R–R, *SDDN*: standard deviation of all normal *RR* intervals recorded in a time interval, expressed in milliseconds, rMSSD: square root of the mean square differences between adjacent normal *RR* intervals, in a time interval, expressed in milliseconds, PNN50: percentage of adjacent IRR with duration differences greater than 50 ms,0V%: percentage of pattern without variation, 1V%: percentage of pattern with one variation, 2LV%: percentage of pattern with two like variation, 2UV%: percentage of pattern with two unlike variations, SE: Shannon entropy, ES: effect size.

**Table 3:** Evaluation of HR kinetics at the beginning and end of the intervention program.

Variables	HIIT-30:30 (*n* = 15)		ES	HIIT-2:2 (*n* = 14)		ES	MICT (*n* = 15)		ES	Group	Time	Group*Time
	Pre	Post		Pre	Post		Pre	Post				
*off*AMP	160.21 ± 16.77	179.73 ± 29.88	−0.37_(medium)_	160.17 ± 25.00	175.36 ± 44.06	−0.20_(small)_	153.21 ± 20.21	172.53 ± 67.22	−0.18_(small)_	0.756	0.030	0.972
*off*TAU	123.19 ± 65.98	72.18 ± 29.20	0.44_(medium)_	131.39 ± 71.05	92.05 ± 33.17	0.33_(medium)_	135.08 ± 99.20	170.44 ± 98.46	−0.17_(small)_	0.012	0.237	0.048
*off*TMR	140.36 ± 75.72	96.95 ± 23.61	0.36_(medium)_	142.44 ± 67.87	136.24 ± 30.84	0.05_(trivial)_	153.00 ± 100.63	224.82 ± 112.65	−0.31_(medium)_	0.002	0.651	0.015

HIIT: high intensity interval training, MICT: moderate intensity continuous training, Group*Time: corresponds to the group × time interaction, Amp: amplitude, TAU: time constant, TMR: average heart rate response time, ES: effect size.

## Data Availability

The raw data supporting the conclusions of this article will be made available by the authors, without undue reservation.

## References

[1] Cho N.H., Shaw J.E., Karuranga S., Huang Y., da Rocha Fernandes J.D., Ohlrogge A.W., Malanda B. (2018). IDF Diabetes Atlas: Global estimates of diabetes prevalence for 2017 and projections for 2045. Diabetes Res. Clin. Pract..

[2] Williams R., Karuranga S., Malanda B., Saeedi P., Basit A., Besançon S., Bommer C., Esteghamati A., Ogurtsova K., Zhang P. (2020). Global and regional estimates and projections of diabetes-related health expenditure: Results from the International Diabetes Federation Diabetes Atlas, 9th edition. Diabetes Res. Clin. Pract..

[3] Shaffer F., Ginsberg J.P. (2017). An Overview of Heart Rate Variability Metrics and Norms. Front. Public Health.

[4] Baldi J.C., Wilson G.A., Wilson L.C., Wilkins G.T., Lamberts R.R. (2016). The Type 2 Diabetic Heart: Its Role in Exercise Intolerance and the Challenge to Find Effective Exercise Interventions. Sports Med..

[5] Stockhorst U., Huenig A., Ziegler D., Scherbaum W.A. (2011). Unconditioned and conditioned effects of intravenous insulin and glucose on heart rate variability in healthy men. Physiol. Behav..

[6] Silva L.R.B.E., Zamunér A.R., Gentil P., Alves F.M., Leal A.G.F., Soares V., Silva M.S., Vieira M.F., Simões K., Pedrino G.R. (2017). Cardiac Autonomic Modulation and the Kinetics of Heart Rate Responses in the on- and off-Transient during Exercise in Women with Metabolic Syndrome. Front. Physiol..

[7] Silva L.R.B., Gentil P., Seguro C.S., de Oliveira G.T., Silva M.S., Zamunér A.R., Beltrame T., Rebelo A.C.S. (2021). High Fasting Glycemia Predicts Impairment of Cardiac Autonomic Control in Adults With Type 2 Diabetes: A Case-Control Study. Front. Endocrinol..

[8] Dhoble A., Lahr B.D., Allison T.G., Kopecky S.L. (2014). Cardiopulmonary fitness and heart rate recovery as predictors of mortality in a referral population. J. Am. Heart Assoc..

[9] Pedersen B.K., Saltin B. (2015). Exercise as medicine—Evidence for prescribing exercise as therapy in 26 different chronic diseases. Scand. J. Med. Sci. Sport..

[10] Rasmussen M.G., Grøntved A., Blond K., Overvad K., Tjønneland A., Jensen M.K., Østergaard L. (2016). Associations between Recreational and Commuter Cycling, Changes in Cycling, and Type 2 Diabetes Risk: A Cohort Study of Danish Men and Women. PLoS Med..

[11] Abrignani M.G. (2018). Physical exercise and risk of arterial hypertension and diabetes mellitus. Let’s move, it is never too late. Eur. J. Prev. Cardiol..

[12] Winding K.M., Munch G.W., Iepsen U.W., Van Hall G., Pedersen B.K., Mortensen S.P. (2018). The effect on glycaemic control of low-volume high-intensity interval training versus endurance training in individuals with type 2 diabetes. Diabetes Obes. Metab..

[13] Cassidy S., Vaidya V., Houghton D., Zalewski P., Seferovic J.P., Hallsworth K., MacGowan G.A., Trenell M.I., Jakovljevic D.G. (2019). Unsupervised high-intensity interval training improves glycaemic control but not cardiovascular autonomic function in type 2 diabetes patients: A randomised controlled trial. Diabetes Vasc. Dis. Res..

[14] Helal L., Umpierre D., Moraes R.S. (2017). High-intensity aerobic interval training improves aerobic fitness and HbA1c among persons diagnosed with type 2 diabetes: Considerations regarding HbA1c starting levels and intervention design. Eur. J. Appl. Physiol..

[15] Kawada T. (2017). Effect of high-intensity aerobic exercise on aerobic fitness and HbA1c in patients with type 2 diabetes. Eur. J. Appl. Physiol..

[16] Støa E.M., Meling S., Nyhus L.K., Glenn S., Mangerud K.M., Helgerud J., Bratland-Sanda S., Støren Ø. (2017). High-intensity aerobic interval training improves aerobic fitness and HbA1c among persons diagnosed with type 2 diabetes. Eur. J. Appl. Physiol..

[17] De Nardi A.T., Tolves T., Lenzi T.L., Signori L.U., Silva A.M.V. (2018). da High-intensity interval training versus continuous training on physiological and metabolic variables in prediabetes and type 2 diabetes: A meta-analysis. Diabetes Res. Clin. Pract..

[18] Guiraud T., Nigam A., Gremeaux V., Meyer P., Juneau M., Bosquet L. (2012). High-intensity interval training in cardiac rehabilitation. Sport. Med..

[19] Gillen J.B., Gibala M.J. (2014). Is high-intensity interval training a time-efficient exercise strategy to improve health and fitness?. Appl. Physiol. Nutr. Metab..

[20] Souza D., Coswig V., de Lira C.A.B., Gentil P. (2020). H″IT″ting the Barriers for Exercising during Social Isolation. Biology.

[21] Kilpatrick M.W., Martinez N., Little J.P., Jung M.E., Jones A.M., Price N.W., Lende D.H. (2015). Impact of high-intensity interval duration on perceived exertion. Med. Sci. Sport Exerc..

[22] Martinez N., Kilpatrick M.W., Salomon K., Jung M.E., Little J.P. (2015). Affective and Enjoyment Responses to High-Intensity Interval Training in Overweight-to-Obese and Insufficiently Active Adults. J. Sport Exerc. Psychol..

[23] Viana R.B., de Lira C.A.B., Naves J.P.A., Coswig V.S., Del Vecchio F.B., Ramirez-Campillo R., Vieira C.A., Gentil P. (2018). Can We Draw General Conclusions from Interval Training Studies?. Sports Med..

[24] Viana R.B., Naves J.P.A., Coswig V.S., de Lira C.A.B., Steele J., Fisher J.P., Gentil P. (2019). Is interval training the magic bullet for fat loss? A systematic review and meta-analysis comparing moderate-intensity continuous training with high-intensity interval training (HIIT). Br. J. Sports Med..

[25] Naves J.P.A., Rebelo A.C.S., Silva L.R.B.E., Silva M.S., Ramirez-Campillo R., Ramírez-Vélez R., Gentil P. (2019). Cardiorespiratory and perceptual responses of two interval training and a continuous training protocol in healthy young men. Eur. J. Sport Sci..

[26] Bento Silva L.R., Viana Gentil P.R., Beltrame T., Basso Filho M.A., Alves F.M., Silva M.S., Pedrino G.R., Ramirez-Campillo R., Coswig V., Silva Rebelo A.C. (2019). Exponential model for analysis of heart rate responses and autonomic cardiac modulation during different intensities of physical exercise. R. Soc. Open Sci..

[27] Borg G.A. (1982). Psychophysical bases of perceived exertion. Med. Sci. Sports Exerc..

[28] Thompson P.D., Arena R., Riebe D., Pescatello L.S. (2013). American College of Sports Medicine ACSM’s New Preparticipation Health Screening Recommendations from ACSM’s Guidelines for Exercise Testing and Prescription, Ninth Edition. Curr. Sports Med. Rep..

[29] Porta A., Faes L., Masé M., D’Addio G., Pinna G.D., Maestri R., Montano N., Furlan R., Guzzetti S., Nollo G. (2007). An integrated approach based on uniform quantization for the evaluation of complexity of short-term heart period variability: Application to 24 h Holter recordings in healthy and heart failure humans. Chaos.

[30] Porta A., Guzzetti S., Montano N., Furlan R., Pagani M., Malliani A., Cerutti S. (2001). Entropy, entropy rate, and pattern classification as tools to typify complexity in short heart period variability series. IEEE Trans. Biomed. Eng..

[31] Imai K., Sato H., Hori M., Kusuoka H., Ozaki H., Yokoyama H., Takeda H., Inoue M., Kamada T. (1994). Vagally mediated heart rate recovery after exercise is accelerated in athletes but blunted in patients with chronic heart failure. J. Am. Coll. Cardiol..

[32] Bearden S.E., Moffatt R.J. (2001). $$\dot{V}o_{2}$$ and heart rate kinetics in cycling: Transitions from an elevated baseline. J. Appl. Physiol..

[33] Wisløff U., Støylen A., Loennechen J.P., Bruvold M., Rognmo Ø., Haram P.M., Tjønna A.E., Helgerud J., Slørdahl S.A., Lee S.J. (2007). Superior cardiovascular effect of aerobic interval training versus moderate continuous training in heart failure patients: A randomized study. Circulation.

[34] Billat L.V. (2001). Interval training for performance: A scientific and empirical practice. Special recommendations for middle- and long-distance running. Part II: Anaerobic interval training. Sports Med..

[35] Cohen J. (1992). A power primer. Psychol. Bull..

[36] Zoppini G., Cacciatori V., Gemma M.L., Moghetti P., Targher G., Zamboni C., Thomaseth K., Bellavere F., Muggeo M. (2007). Effect of moderate aerobic exercise on sympatho-vagal balance in Type 2 diabetic patients. Diabet. Med..

[37] Goulopoulou S., Baynard T., Franklin R.M., Fernhall B., Carhart R., Weinstock R., Kanaley J.A. (2010). Exercise training improves cardiovascular autonomic modulation in response to glucose ingestion in obese adults with and without type 2 diabetes mellitus. Metabolism.

[38] Goit R.K., Pant B.N., Shrewastwa M.K. (2018). Moderate intensity exercise improves heart rate variability in obese adults with type 2 diabetes. Indian Heart J..

[39] Benetos A., Rudnichi A., Thomas F., Safar M., Guize L. (1999). Influence of Heart Rate on Mortality in a French Population: Role of Age, Gender, and Blood Pressure. Hypertension.

[40] Paul L., Hastie C.E., Li W.S., Harrow C., Muir S., John J.M., Dominiczak A.F., McInnes G.T., Padmanabhan S. (2010). Resting heart rate pattern during follow-up and mortality in hypertensive patients. Hypertension.

[41] Zhao M.X., Zhao Q., Zheng M., Liu T., Li Y., Wang M., Yao S., Wang C., Chen Y.M., Xue H. (2020). Effect of resting heart rate on the risk of all-cause death in Chinese patients with hypertension: Analysis of the Kailuan follow-up study. BMJ Open.

[42] Prasada S., Oswalt C., Yeboah P., Saylor G., Bowden D., Yeboah J. (2018). Heart rate is an independent predictor of all-cause mortality in individuals with type 2 diabetes: The diabetes heart study. World J. Diabetes.

[43] Grace F., Herbert P., Elliott A.D., Richards J., Beaumont A., Sculthorpe N.F. (2018). High intensity interval training (HIIT) improves resting blood pressure, metabolic (MET) capacity and heart rate reserve without compromising cardiac function in sedentary aging men. Exp. Gerontol..

[44] Bhatia C., Kayser B. (2019). Preoperative high-intensity interval training is effective and safe in deconditioned patients with lung cancer: A randomized clinical trial. J. Rehabil. Med..

[45] Ramos J.S., Dalleck L.C., Borrani F., Beetham K.S., Mielke G.I., Dias K.A., Wallen M.P., Keating S.E., Fassett R.G., Coombes J.S. (2017). High-intensity interval training and cardiac autonomic control in individuals with metabolic syndrome: A randomised trial. Int. J. Cardiol..

[46] Dall C.H., Snoer M., Christensen S., Monk-Hansen T., Frederiksen M., Gustafsson F., Langberg H., Prescott E. (2014). Effect of high-intensity training versus moderate training on peak oxygen uptake and chronotropic response in heart transplant recipients: A randomized crossover trial. Am. J. Transplant..

[47] Villelabeitia-Jaureguizar K., Vicente-Campos D., Senen A.B., Jiménez V.H., Garrido-Lestache M.E.B., Chicharro J.L. (2017). Effects of high-intensity interval versus continuous exercise training on post-exercise heart rate recovery in coronary heart-disease patients. Int. J. Cardiol..

[48] Qiu S., Cai X., Sun Z., Li L., Zuegel M., Steinacker J.M., Schumann U. (2017). Heart rate recovery and risk of cardiovascular events and all-cause mortality: A meta-analysis of prospective cohort studies. J. Am. Heart Assoc..

[49] Qiu S.H., Xue C., Sun Z.L., Steinacker J.M., Zügel M., Schumann U. (2017). Attenuated heart rate recovery predicts risk of incident diabetes: Insights from a meta-analysis. Diabet. Med..

[50] Besnier F., Labrunée M., Pathak A., Pavy-Le Traon A., Galès C., Sénard J.M., Guiraud T. (2017). Exercise training-induced modification in autonomic nervous system: An update for cardiac patients. Ann. Phys. Rehabil. Med..

[51] Fu Q., Levine B.D. (2013). Exercise and the autonomic nervous system. Handb. Clin. Neurol..

[52] Fisher J.P., Young C.N., Fadel P.J. (2015). Autonomic adjustments to exercise in humans. Compr. Physiol..

